# Ultrasound-Guided Regional Anesthesia Under Sedation for Radical Mastectomy in an SAS Patient: A Case Report

**DOI:** 10.3389/fonc.2021.631003

**Published:** 2021-06-30

**Authors:** Na Zhang, Tingting Wang, Penghui Wei, Jinfeng Zhou, Jianjun Li

**Affiliations:** Department of Anesthesiology, Qilu Hospital (Qingdao), Cheeloo College of Medicine, Shandong University, Qingdao, China

**Keywords:** breast cancer, severe aortic stenosis, anesthesia, regional block, ultrasound guidance

## Abstract

Radical mastectomy is commonly performed under general anesthesia, and regional block is often used as assisted or postoperative analgesia. We herein report a case of successful radical mastectomy with severe aortic stenosis (SAS) by using ultrasound-guided regional anesthesia under sedation. A 66-year-old female with an American Society of Anesthesiology physical status IV; limited functional capacity with <4 metabolic equivalents; a lump (10 cm × 8 cm) in the right breast with skin breakage and infection; and a history of hypertension, diabetes, atrial fibrillation, and SAS, underwent lump-resection and rapid pathological examination by biopsy. Considering a high-risk of significant mortality, we used ultrasound-guided regional block to avoid general anesthesia. We performed the right thoracic paravertebral nerve block (TPVB), subclavicular brachial plexus block, and pectoralis plane block (PECS 1). Patient tolerated the procedure well with no significant hemodynamic changes. Nevertheless, when the axillary lymph nodes were wiped, discharge was observed from the patient’s upper limbs. We inserted the laryngeal mask airway combined with low-dose sevoflurane inhalation sedation. The operation was successfully completed, and the patient was revived with steady hemodynamics and good prognosis. In the present case, radical mastectomy with SAS was performed successfully using ultrasound-guided regional anesthesia under sevoflurane sedation. Despite some potential limitations, this case report can serve as a reference for other anesthetists.

## Introduction

Non-cardiac surgery for patients with severe heart disease is a challenge for both the patient and the anesthesiologist. Therefore, effective anesthesia, sufficient analgesia, and hemodynamic stability should particularly be maintained during surgery for these patients (1). Owing to the definite analgesic effect, the peripheral nerve block techniques may be used in some specific cases as a strategy for avoiding general anesthesia (GA) (2). We herein report a case of successful radical mastectomy with SAS by using ultrasound-guided regional block under sedation. The methods of anesthesia, benefits, and limitations in this case are reviewed and discussed.

## Case Presentation

**Figure 1:**
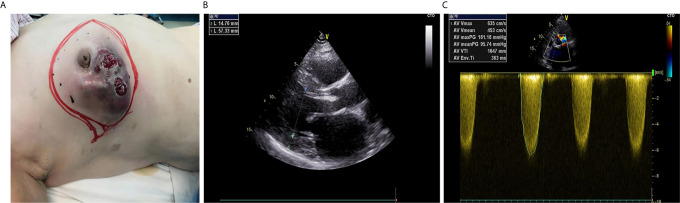
Preoperative information of the patient. **(A)** Appearance of the breast. **(B)** Two-dimensional echocardiography: the enlarged left ventricle and thickened left ventricular wall. **(C)** M-mode echocardiography: accelerated systolic forward flow through aortic valve, increased maximum and average aortic transvalvular pressure difference.

A 66-year-old woman (height: 176 cm, weight: 88 kg) with American Society of Anesthesiology (ASA) physical status IV, history of hypertension, diabetes, SAS, atrial fibrillation, dyspnea, and chest discomfort after labor with <4 metabolic equivalents, was admitted to the hospital with the complaint of a lump (10 cm × 8 cm) in the right breast with skin breakage and infection. The patient had two puncture biopsies of the right breast mass within 2 years, both of which revealed intraductal papilloma. But the lump progressed quickly, covering almost her entire right breast and invading the nipple, and the skin was broken and infected (**Figure 1A**). Therefore, the patient strongly expressed her desire of having breast surgery. Nevertheless, electrocardiogram revealed atrial ectopic rhythm and atrial fibrillation, and echocardiogram demonstrated enlargement of heart, left ventricular myohypertrophia, aortic valve sclerosis, and severe stenosis. The maximum cross valve pressure difference in aortic valve was 114 mmHg and average pressure difference was 72 mmHg, and moderate mitral and tricuspid regurgitation (**Figures 1B, C**). Coronary angiography was performed, and no abnormality was detected. Considering the patient’s severe heart disease and biopsy results in another hospital, she was scheduled for a lump-resection and rapid pathological examination by biopsy.

However, the patient was considered as having high risk for GA. For avoiding GA, a regional anesthesia plan using the ultrasound-guided regional block was proposed to the patient, and anesthesiologic written informed consent was acquired.

On admission to the operating room, standard monitoring was established, and her vitals were recorded as follows: non-invasive blood pressure 155/95 mmHg, heart rate 75 bpm, and SpO_2_ 94%. Then, a patent airway was maintained with oxygen supplementation through the mask at 5 L/min. Vasoactive agents for potential perioperative cardiovascular events, such as esmolol, nicardipine, and norepinephrine, were prepared. Approximately 30 µg dexmedetomidine and 5 µg sufentanil were added into the Murphy’s dropper and dropped slowly. Following this, a skin wheal was raised using 2 ml of 1% lidocaine, and the patient’s radial artery was punctured to measure the invasive arterial pressure.

After sterile technique, the right side thoracic paravertebral nerve block (TPVB) was performed under ultrasound guidance with Philips CX30 Diagnostic Ultrasound System and a high-frequency linear transducer with an in-plane approach. The probe was placed in T4–5 intervertebral space and rotated to locate into the T4–5 paraspinal space. A 22G 80-mm short beveled Stimuplex needle was advanced in plane with the transducer and through the transverse costal ligament. After gentle negative blood aspiration, 30 ml of 0. 375% ropivacaine was injected. In addition, right subclavicular brachial plexus block was performed with 7 ml of 0.375% ropivacaine for blocking the long thoracic nerve. Finally, the ultrasound probe was placed in the outer third of the right subclavian, marked by the thoracic wall branch of the thoracic acromial artery between the pectoralis major and pectoralis minor, and 13 ml of 0.375% ropivacaine was injected to execute the right-side pectoralis plane block (PECS) 1.

No complications were observed. The operation was initiated when the cold sensation was lost, approximately 30 min after the execution of the blocks. The patient tolerated the procedure well and showed no significant hemodynamic changes (blood pressure [BP]: 140/75 mmHg, heart rate [HR]: 60 bpm, SpO_2_: 99%).

**Figure 2:**
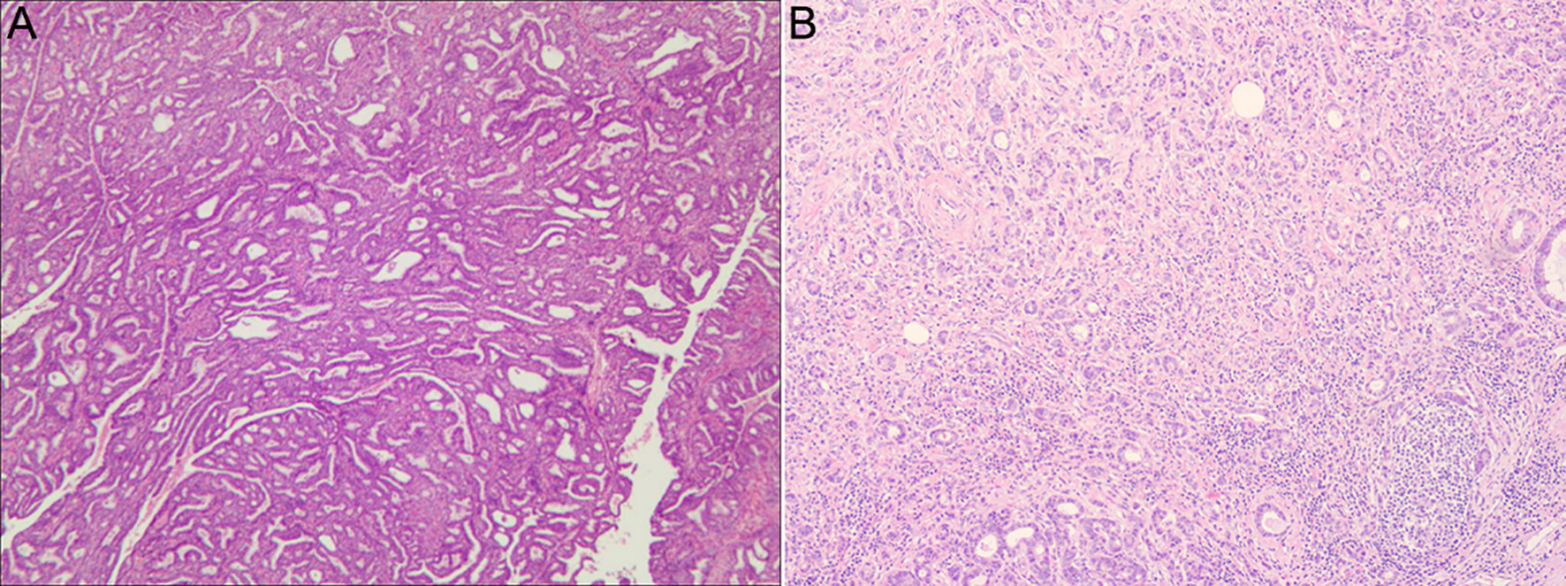
Pathological photographs of the patient. **(A)** Postoperative pathological photograph of the breast. **(B)** Postoperative pathological photograph of the axillary lymph nodes.

However, the surgeon quickly decided to perform modified radical mastectomy upon considering the pathological results (enveloping papillary carcinoma). On wiping the axillary lymph nodes, discharge was observed from the patient’s upper limbs. We decided to insert the laryngeal mask airway combined with sevoflurane inhalation anesthesia, and sedation was achieved with 0.6 MAC sevoflurane without using opioids. During this period, the patient had stable vital signs with BP: 110/70 mmHg, HR: 55 bpm, and SpO_2_: 99%. In addition, the patient breathed spontaneously for the perioperative period. Approximately half an hour later, the axillary nodes were dissected, and laryngeal mask was pulled out for narrowing the duration of GA. The operation was successfully completed. The operation time was 2.5 h, and the intraoperative blood loss was 50 ml and urine volume was 300 ml. The patient received 1,300 ml of Ringer’s solution during surgery. And the patient was revived with steady hemodynamics. We maintained communication with the patient from the regional block process to inhalation-anesthesia, and the patient cooperated without any complaint of discomfort. Postoperatively, the patient confirmed that she was satisfied with the anesthesia process. The postoperative pathological findings revealed an enveloping papillary carcinoma with infiltrating papillary carcinoma and (9/12) lymph node metastases (**Figures 2A, B**). The patient was shifted to the cardiac surgery intensive care unit following the procedure, then shifted back to the general ward after 2 days and was discharged after 11 days. A 3-month follow-up revealed that the patient was in fair condition. Chemotherapy had been initiated, and cardiac surgery was planned.

**Figure 3:**
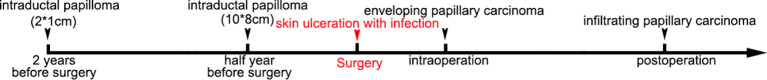
Timeline of the patient’s medical history. Two years before surgery, the mass in the right breast was approximately 2 cm × 1 cm, and the puncture result was intraductal papilloma. The mass developed to be a size of 10 cm × 8 cm 18 months later, while the puncture result was intraductal papilloma. Subsequently, the skin was broken and severely infected. The intraoperative pathology was enveloping papillary carcinoma. The postoperative pathology was enveloping papillary carcinoma with infiltrating papillary carcinoma and (9/12) lymph node metastases.

A timeline of the patient’s medical history is presented in **Figure 3**.

## Discussion

Herein, we report the successful use of ultrasound-guided regional block for breast surgery in a patient with SAS. As per by the American College of Cardiology/American Heart Association, aortic stenosis is graded as when at least one of the following three parameters are present: aortic valve area ≤1 cm^2^, peak velocity >4 m/s, mean gradient ≥40 mmHg on echocardiography; the patient is then regarded as high risk for non-cardiac surgery (3). In addition, patients showing symptoms are generally considered to be at a greater risk compared with those without symptoms. Recent studies have demonstrated that SAS is associated with an increased risk of major adverse cardiovascular events (4, 5). All major guidelines suggest postponement of any major non-cardiac surgery until AS is addressed by aortic valve replacement or percutaneous interventions. The European Society of Cardiology/the European Society of Anesthesiology guidelines consider symptoms as critical factors for deciding whether elective non-cardiac surgery can be performed (1). However, our breast cancer patient, with dyspnea and chest discomfort after labor, was contraindicated for aortic valve replacement surgery. While the damaged skin is prone to serious infection, cardiac surgery risks the spread of malignant tumors. This posed several hemodynamic challenges for the anesthesiologist while scheduling the patient for breast surgery.

Yearly, almost half of the newly diagnosed breast cancer cases occur in the aged-population (≥65 years), and most have several complications (6). Therefore, peri-operative morbidity and mortality in association with breast cancer should not be underestimated. Thus, we recorded the details of the anesthesia procedure for the patient combined with breast cancer and SAS in hopes of providing a reference for other anesthesiologists.

**Figure 4:**
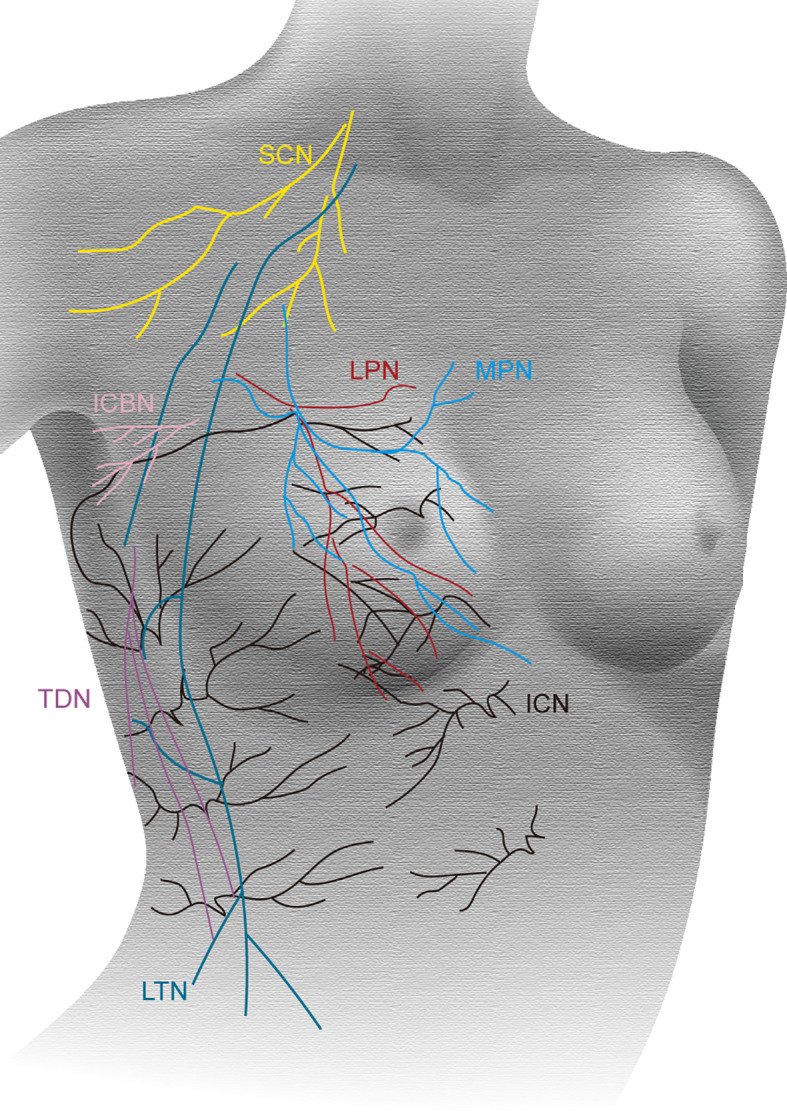
Innervation of the breast. The supraclavicular nerves (SCN, yellow); anterior and lateral branches of the intercostal nerves (ICN, black); lateral pectoral nerve (LPN, red); medial pectoral nerve (MPN, blue); intercostobrachial nerve (ICBN, pink); long thoracic nerve (LTN, green); thoracodorsal nerve (TDN, purple).

Although radical mastectomy is commonly performed under GA, in our patient with concomitant SAS, there was a risk of significant mobility and mortality. During GA, a fall in vascular resistance results in hypotension owing to inadequate compensatory increase in cardiac output due to a stenotic valve. In turn, hypotension reduces coronary perfusion. This further leads to myocardial ischemia and death (3). Furthermore, the use of muscle relaxant drugs and positive airway pressure in the intubated patient could theoretically cause an even greater hemodynamic change, deleterious to the patient with AS (7). Neuraxial anesthesia is rarely used for patients with SAS, owing to its sympathetic blockade effect that can potentially cause loss of vascular tone and cardiac output (8). Regional anesthesia, such as TPVB, is a popular technique in breast surgery. New fascial plane blocks, including the PECS 1 and PECS 2, and serratus plane block, are also alternative for the chest wall analgesia (**Figure 4**). There are several reports about breast surgery under regional anesthesia. Most of them involve sedation using propofol and/or remifentanil target-controlled infusion, providing assisted or postoperative analgesia (2, 9). In addition, patients who had a total mastectomy with axillary node clearance require conversion to GA (6).

For maintaining cardiovascular stability and avoiding hypotension and ischemia, we choose regional anesthesia. Considering enhanced hemodynamic changes of propofol, remifentanil, and muscle relaxant drugs, we performed sevoflurane inhalation under spontaneous breathing. In addition, after the wiping of axillary node, pulling out the laryngeal mask immediately shortens the duration of GA. To avoid hypertension and tachycardia, we performed the combined nerve block under sufficient local anesthesia. Continuous invasive arterial pressure monitoring was performed during surgery. Hemodynamic monitoring system and transesophageal echocardiography (TEE) were available in the operating room as backup. The whole process is performed successfully with a stable hemodynamics with high levels of patient acceptability and surgeon satisfaction.

There are some limitations in our case report. First, the effect of nerve block was not complete. The skin of the axilla was supplied by the intercostobrachial nerve and medial brachial cutaneous nerve, therefore, TPVB combined PECS 2 and supraclavicular nerve block might have been better. Secondly, there was a risk of poisoning, owing to the greater amount of local anesthetics. Third, recent evidence indicates that volatile anesthetics have a relationship with cancer recurrence (10). Though, the duration of inhalation anesthesia was short. In addition, regional anesthesia may reduce the incidence of cancer recurrence through opioid-sparing effects or direct mechanisms. Fourth, the patient may benefit from a proper hemodynamic monitoring system such as LIDCO, PICCO, and TEE, though she was hemodynamically stable during anesthesia. Finally, we have reported short-term outcomes; however, follow-up is required for long-term outcomes.

## Conclusions

In conclusion, despite these potential limitations, our case report is a novel and successful exploration where the nerve block plays a major role in radical mastectomy. Thus, this case report can provide reference for other anesthetists.

## Data Availability Statement

The original contributions presented in the study are included in the article/supplementary material. Further inquiries can be directed to the corresponding authors.

## Ethics Statement

Written informed consent was obtained from the individual(s) for the publication of any potentially identifiable images or data included in this article.

## Author Contributions

PW, JL, and JZ performed the anesthesia and revised the manuscript. NZ, TW, and PW contributed to writing and collection of data. All authors contributed to the article and approved the submitted version.

## Funding

This work was supported by grants from the Natural Science Foundation of Shandong Province (ZR2020QH291 and ZR2020MH126), the Key Research and Development Plan of Shandong Province (2019GSF108228), the Qingdao Key Health Discipline Development Fund (2019), and the Qingdao Outstanding Health Professional Development Fund (2019). The funders have no role in case design, data collection, analysis, and manuscript preparation.

## Conflict of Interest

Authors declare that the research was conducted in the absence of any commercial or financial relationships that could be construed as a potential conflict of interest.
